# A comparison of Monte Carlo sampling methods for metabolic network models

**DOI:** 10.1371/journal.pone.0235393

**Published:** 2020-07-01

**Authors:** Shirin Fallahi, Hans J. Skaug, Guttorm Alendal

**Affiliations:** Department of Mathematics, University of Bergen, Bergen, Norway; Universidad Rey Juan Carlos, SPAIN

## Abstract

Reaction rates (fluxes) in a metabolic network can be analyzed using constraint-based modeling which imposes a steady state assumption on the system. In a deterministic formulation of the problem the steady state assumption has to be fulfilled exactly, and the observed fluxes are included in the model without accounting for experimental noise. One can relax the steady state constraint, and also include experimental noise in the model, through a stochastic formulation of the problem. Uniform sampling of fluxes, feasible in both the deterministic and stochastic formulation, can provide us with statistical properties of the metabolic network, such as marginal flux probability distributions. In this study we give an overview of both the deterministic and stochastic formulation of the problem, and of available Monte Carlo sampling methods for sampling the corresponding solution space. We apply the ACHR, OPTGP, CHRR and Gibbs sampling algorithms to ten metabolic networks and evaluate their convergence, consistency and efficiency. The coordinate hit-and-run with rounding (CHRR) is found to perform best among the algorithms suitable for the deterministic formulation. A desirable property of CHRR is its guaranteed distributional convergence. Among the three other algorithms, ACHR has the largest consistency with CHRR for genome scale models. For the stochastic formulation, the Gibbs sampler is the only method appropriate for sampling at genome scale. However, our analysis ranks it as less efficient than the samplers used for the deterministic formulation.

## Introduction

Cell metabolism involves many chemical reactions, catalyzed by thousands of enzymes, and is often represented as metabolic networks [1]. The dynamics of a metabolic network, consisting of *m* metabolites and *n* reactions, can be mathematically modelled by a system of Ordinary Differential Equations (ODEs) written in short form as

$$\begin{array}{c} \frac{dx}{dt}=Sv\left(x\left(t\right),\alpha ,t\right). \end{array}$$ (1)

Here, $x\in R^{m}$ is a vector containing of metabolite concentrations, $\alpha \in R^{k}$ is a vector of parameters, $S\in R^{m\times n}$ is the stoichiometric matrix, i.e. a matrix representation of the network, and $v\left(x,\alpha ,t\right)\in R^{n}$ are the flux rates in the *n* reactions [2].

The stoichiometric matrix *S* is constructed so that element *S*_*ij*_ is positive (negative) if metabolite *i* is created (consumed) by reaction *j*, represented by the flux rate *v*_*j*_, and is assumed constant. A challenge is to establish models of the different flux rates, in general nonlinear in **x**, and to estimate the *k* parameters in **α** through in-vivo and in-vitro experiments. The non-linearity of the ODE system also makes the system susceptible to chaotic behavior, bifurcation and sensitivity to parameter values [3].

In Flux Balance Analysis (FBA) [4] the model system is assumed to be in a steady state

$$\begin{array}{c} \frac{dx}{dt}=S\;v=0, \end{array}$$ (2)

i.e. the problem goes from being a set of differential equations in **x** to become an algebraic problem, with the flux rates **v** as unknowns. Often the flux rates are constrained with upper and lower bounds

$$\begin{array}{c} v^{lb}\leq v\leq v^{ub}. \end{array}$$ (3)

However, since a typical metabolic network has fewer metabolites than reactions, i.e. *m* < *n*, the system in Eqs (2) and (3) is in general undetermined. The system might have many feasible solutions in a closed convex polytope, the n-dimensional analogue to the three dimensional polyhedron, formed by the intersection of the kernel of *S* and the linear inequalities in Eq (3) [5]. A unique solution might be found by introducing an objective function which aims to optimize some biological functionality, for example maximizing cell growth rate or ATP production of an organism [2]. A challenge in FBA is to choose the most appropriate objective function.

An alternative to FBA, which avoids the need to specify an objective function, is to sample (uniformly) from the flux polytope defined by Eqs (2) and (3). The solution space can then be characterized statistically from the set of sampled **v** vectors in terms of a probability density function (pdf), which we denote by *p*(**v**) [6]. We will distinguish between a *deterministic* and *stochastic* formulation of given metabolic model and the associated flux measurements. The stochastic formulation is more flexible in that it can account for measurement error and allows relaxation of the steady state condition in Eq (2).

**Fig 1:**
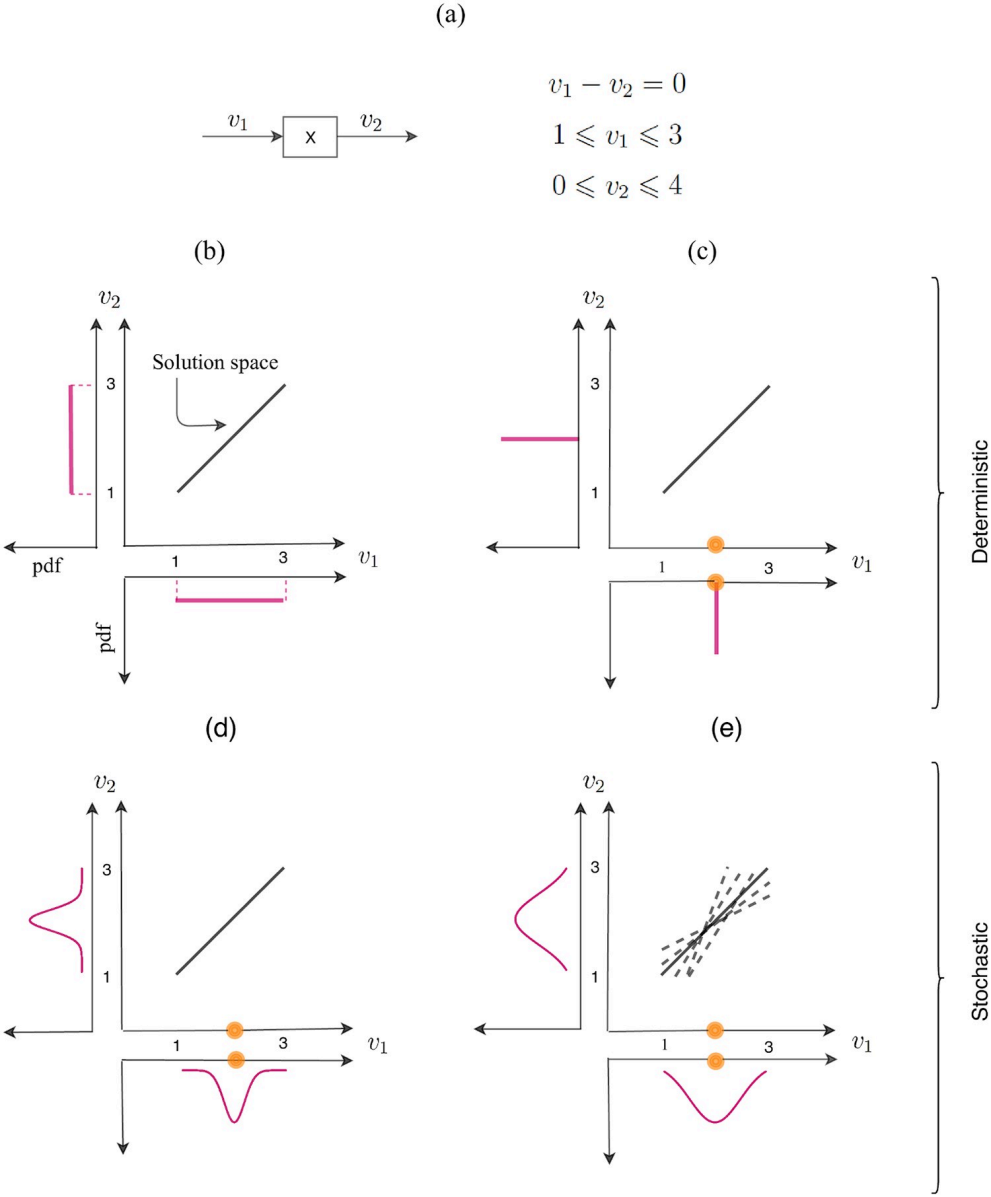
Solution space and sampling pdf *p*(*v*) (pink curve) under different experimental setups. (a): Example metabolic network and corresponding mathematical model. (b): Deterministic formulation without measurements. (c) and (d): Flux measurement of *v*_1_ (orange circle) available in deterministic setup (c) and stochastic setup (d). (e): Relaxed steady state assumption and flux measurement of *v*_1_ in stochastic setup.

Fig 1 illustrates the key concepts used in this paper. The simple metabolic network consists of a single input flux, *v*_1_, *m* = 1 metabolite, and a single output flux *v*_2_, i.e. *n* = 2 fluxes in total. The resulting constrained steady state equation is given in Fig 1a). Panel b) shows the polytope representing the solution space, which in this case is a line segment in the *v*_1_-*v*_2_ plane. Panel b) also shows the uniform pdf’s *p*(*v*_1_) and *p*(*v*_2_) indicating that all flux values in the feasible intervals are “equally probable”.

Fig 1 illustrates how the deterministic and stochastic frameworks differ in the way they incorporate flux measurements. In the deterministic case (Panel c), fixing *v*_1_ experimentally uniquely determines *v*_2_. Both pdf’s collapse to point masses, and all other a-priori feasible values have zero probability. In a stochastic framework (Panel d), on the other hand, the uncertainty in the measurement of *v*_1_ can be taken into account. When this uncertainty is combined with constraints imposed by the polytope, the resulting pdf’s *p*(*v*_1_) and *p*(*v*_2_) are non-degenerate as shown in Panel d), and displays the marginal likelihood of each feasible flux value.

Another limiting assumption of the deterministic formulation is the exact steady state assumption. This assumption is not always realistic and should be relaxed to have a model compatible with the stochastic nature of biological networks [7, 8]. In Fig 1e), we relax the steady state assumption (2), while still incorporating the uncertain measurement *v*_1_. This leads to wider pdf’s (Panel e versus d), and the solution polytope is not necessarily convex any more.

For genome-scale metabolic models the dimension (*n*) of the polytope formed by Eqs (2) and (3) is typically high and deterministic sampling from such polytopes is challenging [9]. Hence, Monte Carlo (MC) approximations are often used [10]. In Wiback et al. [11] a MC rejecting sampling algorithm was used to sample low dimensional polytopes. However, this algorithm becomes inefficient when *n* is large, so a more commonly used algorithm is the hit-and-run (HR) [12, 13], which is a Markov Chain Monte Carlo (MCMC) method. Almaas et al. [14] originally applied the HR algorithm to the bacterium Escherichia coli metabolic network. The algorithm efficiently samples from the solution space as long as the polytope is isotropic in scales of the fluxes, i.e. being independent on direction in the high dimensional sample space.

High dimensional polytopes that are very narrow in some directions are difficult to sample properly. To cope with this anisotropy problem, the artificial centering hit-and-run (ACHR) algorithm has been developed [15]. The ACHR algorithm and an algorithm based on ACHR, known as optimized general parallel sampler (OPTGP) [16], are widely used to sample the solution space of metabolic models. However, both samplers suffer from convergence problems due to the non-Markovian nature of ACHR [17]. The ACHR algorithm is implemented in both the COnstrained Based Reconstruction and Analysis (COBRA) toolbox [18] (in Matlab) and COBRApy (in Python). The OPTGP is available only in COBRApy. Recently, rounding procedures have been proposed to remove the heterogeneity issue of the solution space, and then a modified version of HR is used [17, 19]. Coordinate hit-and-run with rounding (CHRR) [19] is also implemented in the COBRA toolbox. The algorithms mentioned so far are designed to sample the polytope formed by a deterministic formulation of the model (Fig 1b and 1c). The run time and convergence of the two ACHR based algorithms and CHRR are compared using three constraint-based models in the study by Herrmann et al. [6].

In the study by Van den Meersche et al. [20] a general framework to solve a linear inverse problem using a MCMC algorithm is presented. The suggested framework can be used to sample the solution space of a metabolic network model which is constructed to encode an exact steady state assumption, bounded fluxes and flux observations with related experimental noise (Fig 1d). A function is available in the *limSolve* R package [21] to perform the sampling in this framework.

Another option is to relax the steady state constraint in Eq (2) while including the flux data and corresponding noise (Fig 1e). Considering these assumptions, a statistical model using Bayesian framework has been introduced by Heinonen et al. [22], and a truncated multivariate normal (*TMVN*) posterior distribution for the fluxes has been presented. Efficient sampling from a truncated multivariate normal distribution is a challenging task, and often Gibbs sampling is applied [23]. The Bayesian metabolic flux analysis (BMFA) is implemented in the COBRA toolbox by Heinonen et al. [22].

To our knowledge this is the first time that both available deterministic and stochastic frameworks are reviewed and corresponding sampling algorithms are compared to each other. In this study we have evaluated ACHR, OPTGP and CHRR algorithms which are appropriate for the deterministic formulation. Even if we use different criteria than the ones used by Herrmann et al. [6] our results are in good agreement with their findings. In addition, we have evaluated sampling algorithms *xsample()* and Gibbs which are related to the stochastic formulation. These algorithms have not been discussed by Herrmann et al. [6].

First we give an overview of available MC sampling algorithms for the different cases presented in Fig 1, and discuss their pros and cons. Then, an assessment of algorithms in terms of convergence, consistency and efficiency is given. We conclude the paper with a discussion on which framework and sampling algorithm might be better to use considering restrictions in the model and level of uncertainty for available flux measurements.

## Survey of sampling algorithms

Below follows a brief description each of the algorithms included in this study, cast in a common notation. For more details the reader is referred to the background papers.

### Deterministic formulation

We begin by describing the standard hit-and-run (HR) algorithm to sample from a convex set. We then review HR related algorithms to approximate uniform sampling from a convex polytope, which is a convex set of points, constructed by the exact steady state in Eq (2) and the capacity constraints in Eq (3) on metabolic fluxes.

#### The Hit-and-Run sampling algorithm (HR)

The standard HR algorithm collects samples from a given *N* dimensional convex set *P* by choosing an arbitrary starting point **v**^(0)^ ∈ *P*, setting *a* = 0 where *a* is the iteration number and going iteratively through three steps:

1. choosing an arbitrary direction ***θ***^(*a*)^ uniformly distributed on the boundary of the unit sphere in $R^{N}$;
2. finding the minimum (maximum) value of λ∈R denoted by λ_*min*_ (λ_*max*_) such that **v**^(*a*)^ + λ***θ***^(*a*)^ ∈ *P* and choose a random step size λ^(*a*)^ ∈ [λ_*min*_, λ_*max*_];
3. generating a new sample **v**^(*a*+1)^ = **v**^(*a*)^ + λ^(*a*)^ ***θ***^(*a*)^ by taking a step of size λ^(*a*)^ from the current sample **v**^(*a*)^ in the direction ***θ***^(*a*)^ and then set *a* = *a* + 1.

The HR technique is a MCMC approach since it generates a new sample by using only the current sample point, which is the definition of the Markov property. Convergence to the target distribution is guaranteed for a MCMC sampling approach, see for instance [24].

The simple HR algorithm performs effectively in a high dimensional space as long as the solution space is isotropic. A bottleneck of the standard HR is the diffusion in the presence of narrow corners in the solution space due to tightly constrained fluxes. In narrow regions HR has to take small steps and consequently the new sample is close to the previous one. This prevents the sampler to perform a full exploration of the solution space of an irregular shape in a finite time, and is known as *slow mixing*.

#### Artificial Centering Hit-and-Run (ACHR)

The artificial centering hit-and-run (ACHR) was proposed by Kaufman et al. [15] to overcome the problem of slow mixing. In a highly heterogeneous solution space a uniform direction choice on the boundary of the unit sphere is a poor choice. The core idea of the ACHR is to use optimal direction choices in HR to allow for larger steps along the elongated directions. In each iteration the sampler tries to approximate a center for the space by computing the mean of all the samples generated so far for each coordinate. Then it chooses randomly a sample from all the samples generated and find a new direction by normalizing the difference between the selected sample and the current approximated center. Considering an arbitrary starting point **v**^(0)^ ∈ *P*, a number of warm up samples *M*_*warm*_ ≥ *N*, setting *a* = 0 and an initial center $\hat{c}=v^{\left(0\right)}$, ACHR generates samples iteratively by performing four steps:

1. generate a direction: if *a* < *M*_*warm*_ (warm up phase), select a direction ***θ***^(*a*)^ as in the standard HR approach. Otherwise (main phase) choose a number *i* uniformly distributed on {0, 1, …, *a*} and compute a direction $\theta^{\left(a\right)}=\frac{v^{\left(i\right)}-\hat{c}}{∥v^{\left(a\right)}-\hat{c}∥}$;
2. choose a random step size λ^(*a*)^ as in the standard HR;
3. generate a new sample **v**^(*a*+1)^ = **v**^(*a*)^ + λ^(*a*)^ ***θ***^(*a*)^ and then set *a* = *a* + 1;
4. update the artificial center by setting $\hat{c}=\frac{a\hat{c}+v^{\left(a\right)}}{a+1}$.

In each iteration of ACHR in the main phase, the direction is dependent on all previous iterates and directions and this makes the sampler a non-Markovian algorithm. Therefore it is not guaranteed that the sequence of iterates converges toward the target distribution.

For genome scale metabolic models, this algorithm might perform slow to sample the polytope formed by the solution space. To make the sampling process faster, an algorithm named the optimized general parallel sampler (OPTGP) was proposed by Megchelenbrink et al. [16]. In this algorithm the flux through each reaction is maximized and minimized to generate the 2*n* warm-up points. From warm up points, this algorithm generates multiple short chains in parallel using the approximated center as in ACHR and it takes only the *k*^*th*^ point of the chain as a sample point [16]. In the study by Megchelenbrink et al. [16] it has been shown that the OPTGP performs more efficient than the ACHR by generating samples with higher randomness in a shorter time. Clearly, the ACHR is at the core of the OPTGP and this leads to a non-Markovian algorithm. Even though both algorithms are commonly used in the literature, both of them suffer from convergence problems [17].

#### Coordinate Hit-and-Run with Rounding (CHRR)

As mentioned, the performance of the HR algorithm can be strongly affected by irregularity in the shape of the polytope *P* representing the solution space, known as ill-conditioning. Suppose *R*_*b*_ is the radius of the biggest ball that can be placed inside the polytope and *R*_*s*_ is the radius of the smallest ball inscribing the polytope. The time a sampling algorithm takes to converge to the target distribution is called the mixing time *τ* and in Lovász et al. [25] it has been shown that the mixing time of the HR algorithm scales by

$$\begin{array}{c} \tau ≃O(N^{2}\frac{R_{s}^{2}}{R_{b}^{2}}), \end{array}$$ (4)

where *N* is the dimension of the polytope. The degree of ill-conditioning for the sampling problem is measured by *R*_*s*_/*R*_*b*_, known as the sandwiching ratio of the body. This ratio depends on the orders of magnitude of the flux scales and in genome scale problems this number can reach 10^5^ which indicates very high irregularity of the polytope to be sampled [17].

To reduce the sandwiching ratio and eliminate ill-conditioning, an approach is presented in Haraldsdottir et al. [19] that consists of two steps; rounding and sampling. In the rounding phase a maximum volume inscribed ellipsoid is built, based on the presented algorithm in Zhang et al. [26], to match closely the heterogeneous polytope. Then the polytope is rounded through transforming the inscribed ellipsoid to a unit ball. A variant of HR algorithm known as coordinate hit-and-run (CHR) [27] is used to sample from the rounded polytope. In the CHR algorithm the direction *θ*^(*a*)^ is selected randomly along the coordinate directions instead of picking randomly from the unit sphere in $R^{N}$. Otherwise the CHR algorithm operates similar to the HR. After running the CHR algorithm the sampled points are transformed back to the original space through an inverse transformation. Since the CHRR uses CHR Markov chain for sampling purpose, its convergence to the target distribution is guaranteed in contrast to ACHR based algorithms [28].

### Stochastic formulation

In this part we review the studies of Van den Meersche et al. [20] and Heinonen et al. [22] in which statistical frameworks have been proposed to analyze metabolic fluxes while integrating flux measurements with their noise in the formulation and relaxing the steady state assumption in Eq (2). To our knowledge, these two studies are the only studies presenting sampling algorithms applicable at genome scale.

#### Sampling linear inverse problems (*xsample()*)

In the deterministic formulation represented by Eqs (2) and (3) if the experimental values for some of the fluxes are available, they are integrated in the formula by fixing the fluxes at the given values. However, we do not account for the uncertainty of the flux measurements in the equations if we fix the fluxes at their measured values and this might result in overconfidence in outcomes and conclusions. In Van den Meersche et al. [20] the uncertainties corresponding to the experimental values were included in the Eqs (2) and (3) by adding a noise term to the algebraic equation

$$\begin{array}{c} Av=b+\epsilon , \end{array}$$ (5)

where the data vector is denoted by **b** and corresponding uncertainties are encoded by ***ϵ*** ∼ *N*(**0**, Σ). The diagonal matrix Σ = *diag*(*σ*_1_, …, *σ*_*n*_) represents the variances of flux data. The matrix *A* is a diagonal matrix where *a*_*ii*_ is one in the presence of data for *v*_*i*_ and otherwise zero. The model describes the exact steady state phase of the network considering the limited capacity of the fluxes and it also accounts for the available flux measurements with their experimental noise.

Van den Meersche et al. [20] provided a function named *xsample()* in R [29] to produce a set of samples of fluxes **v** in this framework. The function produces the samples by carrying out a two-staged process. First the equality constraint *S* **v** = **0** is eliminated since all solutions **v** for this system of equations can be written as

$$\begin{array}{c} v=Gu \end{array}$$ (6)

where $G\in R^{n\times (n-r_{s})}$ is an orthonormal matrix formed by the basis for the null space of *S* (*r*_*s*_ is the rank of *S*). The linearly dependent variables $v\in R^{n}$ are transformed to linearly independent variables $u\in R^{n-r_{s}}$. The constraints in terms of **u** are

$$\begin{array}{c} AGu=b+\epsilon \end{array}$$ (7)

$$\begin{array}{c} v^{\text{lb}}\leq Gu\leq v^{\text{ub}}. \end{array}$$ (8)

In the second stage the variables **u** are sampled from a proposed *TMVN* distribution with probability density function

$$\begin{array}{c} p\left(u\right)\propto \{\begin{array}{cc} e^{-\frac{1}{2}{\left(AGu-b\right)}^{T}\Sigma^{-1}\left(AGu-b\right)} & \text{if}\;v^{\text{lb}}\leq Gu\leq v^{\text{ub}}\\ 0 & \text{otherwise} \end{array}. \end{array}$$ (9)

To sample from this distribution, the *xsample()* applies the Metropolis algorithm [30]. The *xsample()* function in R allows to examine three different jump (proposal) algorithms. However, here we discuss only one of them named the mirror algorithm which has been found to perform more efficient for high-dimension problems [20]. This algorithm uses the inequality constraints in Eq (8) as reflective planes. Assume **u**^(*a*)^ is a feasible sample and a new point will be drawn

$$\begin{array}{c} u_{0}^{\left(a+1\right)}\in N\left(u^{\left(a\right)},\Omega \right) \end{array}$$ (10)

where the normal distribution is in the unrestricted space with mean **u**^(*a*)^ and a set of fixed standard deviations collected in the diagonal matrix $\Omega =diag(\omega_{1},\ldots ,\omega_{n-r_{s}})$. If the point $u_{0}^{\left(a+1\right)}$ fulfills all inequalities in Eq (8), it is accepted as the point **u**^(*a*+1)^ to be evaluated by the acceptance ratio test in the Metropolis algorithm [40]. But if the point $u_{0}^{\left(a+1\right)}$ violates some inequalities, it is mirrored consecutively in the hyperplane formed by violated inequalities [20]. Then the resulting point **u**^(*a*+1)^ satisfies all inequalities and will be evaluated through the acceptance ratio test to be accepted or rejected.

The diagonal elements of the matrix Ω are the jump lengths of the Markov Chain. The jump lengths define the step lengths taken and they determine the distance covered within the solution space in one iteration and also the number of reflections in the solution space boundaries. Due to this the jump lengths have a significant influence on the efficiency of this algorithm.

#### Bayesian Metabolic Flux Analysis (BMFA)

So far we have considered frameworks in which the metabolic network is constrained to the exact steady state. In 2016, it was shown that metabolites can accumulate or deplete in a metabolic network [8] and recently MacGillivray et al. [7] studied metabolic networks under the relaxed steady state assumption through the so-called RAMP model. They have presented an argument that the exact steady state constraint (Eq (2)) on the fluxes should be relaxed to be in agreement with the stochastic nature of a cell. In 2019, a statistically relaxed steady state model was presented in Heinonen et al. [22]

$$\begin{array}{c} Sv=0+\beta , \end{array}$$ (11)

where ***β*** ∼ *N*(**0**, **Γ**) is a vector of disturbances around the steady state assumption *S* **v** = **0**. The allowed variances around the steady state are collected in the diagonal matrix *Γ* = *diag*(**γ**) = *diag*(*γ*_1_, …, *γ*_*m*_). Note that by considering very small variances, **γ** → 0, the model will be compatible with the strict steady state case.

Heinonen et al. [22] implemented Eq (11) in a Bayesian framework in which multivariate Gaussian priors for fluxes were assumed. The prior mean for a flux was set to zero or to the closet value to zero considering the flux upper and lower bounds. The prior variances as a hyperparameter defines the a priori values a flux can take. A *TMVN* distribution *TMVN*(**μ**, *C*, **v**^*lb*^, **v**^*ub*^) was proposed as the target distribution from which fluxes **v** were sampled. For sampling purpose, Heinonen et al. [22] used the Gibbs algorithm [31], which is a MCMC algorithm suitable for Bayesian models. Detailed formulas for the mean vector **μ** and the covariance matrix *C* can be found in [22].

In Heinonen et al. [22], the flux variables **v** were first transformed to uncorrelated variables $\tilde{v}=L^{-1}\left(v-\mu \right)$ using a Cholesky decomposition of the covariance matrix *C* = *LL*^*T*^ to make the sampling process more efficient. Thereafter the problem was converted to sample $\tilde{v}$ from the distribution $TMVN(0,I,{\tilde{v}}^{lb},{\tilde{v}}^{ub})$ where *I* is the identity matrix, ${\tilde{v}}^{lb}=v^{lb}-L\mu$ and ${\tilde{v}}^{ub}=v^{ub}-L\mu$. In the Gibbs algorithm an initial sample point ${{\tilde{v}}_{j}}^{\left(0\right)}$ is drawn from the Gaussian prior distribution for *j* = 1…*n*. Then, at each iteration the algorithm cyclically (*j* = 1…*n*) draws ${\tilde{v}}_{j}$ from the conditional posterior density $p({\tilde{v}}_{j}|{\tilde{v}}_{-j})$, where ${\tilde{v}}_{-j}$ is a vector including all fluxes except the flux ${\tilde{v}}_{j}$. Using properties of the *TMVN* distribution, it can be shown that these conditional distributions again are within the *TMVN*, and Heinonen et al. [22] has provided closed form expressions for the upper and lower bounds ${\tilde{v}}^{lb}$ and ${\tilde{v}}^{ub}$.

A summary of the sampling algorithms and their main characteristics are presented in the Table 1.

**Table 1:** A summary of sampling algorithms and their main characteristics.

Sampling algorithm	Programming language	Convergence guaranteed?	Relevant formulation
ACHR	Matlab/Python	No	Deterministic
OPTGP	Python	No	Deterministic
CHRR	Matlab	Yes	Deterministic
Gibbs/BMFA	Matlab	Yes	Stochastic
*xsample()*	R	Yes	Stochastic

## Experimental setup and implementation

The four sampling algorithms (ACHR, OPTGP, CHRR and Gibbs) were applied to sample from ten metabolic models, which were obtained from the BiGG database [32]. The sampling algorithms were applied on one core model (E. coli core) and nine genome scale metabolic models with the number of fluxes ranging from *n* = 95 to *n* = 3741. The *M* = 20, 000 samples were generated for each flux in each model, with a thinning parameter of 1000 in each sampling algorithm where we kept every 1000 draw from the target distribution and discarded the rest.

The OPTGP and Gibbs algorithm sampled from the full models, while ACHR and CHRR sampled from reduced versions of the models, obtained as follow. The upper and lower bounds on the fluxes (**v**^*lb*^ and **v**^*ub*^) were changed to the minimum and maximum achievable flux values computed through flux variability analysis [33]. Then, the model was reduced by discarding the reactions which could not carry any flux (null reactions with maximum and minimum achievable values less than a threshold). Table 2 shows summary statistics for each metabolic model, including the number of reactions before (*n*) and after (*n*_*red*_) reduction. Also shown are AFR values, i.e. Average Flux Range of the full models calculated by $AFR=1/n\sum_{j=1}^{n}\left(v_{j}^{ub}-v_{j}^{lb}\right)$.

**Table 2:** Constraint-based metabolic models and run times (min) for different sampling algorithms. The *m*, *n*, *n*_*red*_ denote the number of metabolites, reactions of the full model and of the reduced model, respectively. The AFR is the Average Flux Range of the full model. The 20, 000 samples for each flux in each metabolic model were drawn on an Intel Core i7 at 2.5 GHz. In all sampling algorithms the thinning parameter was set to 1000.

Network					Run time			
Model	*m*	*n*	*n*_*red*_	AFR	ACHR (Deterministic)	OPTGP (Deterministic)	CHRR (Deterministic)	Gibbs/BMFA (Stochastic)
E. coli core	72	95	87	1474	68.78 min	14.81 min	6.17 min	69.96 min
iAB_RBC_283	342	469	453	1080	99.67 min	18.53 min	9.46 min	1148.50 min
iLJ478	570	652	380	1292	91.08 min	19.83 min	7.64 min	1884.00 min
iSB619	655	743	450	1267800	96.09 min	20.83 min	9.55 min	2173.50 min
iHN637	698	785	522	1257	103.38 min	22.13 min	7.85 min	2483.50 min
iAT_PLT_636	738	1008	1008	1444	132.55 min	27.56 min	13.81 min	2244.80 min
iJN746	907	1054	652	1329200	116.40 min	24.80 min	10.67 min	3179.70 min
iSDY_1059	1888	2539	1502	1248	148.64 min	40.15 min	18.40 min	17393.00 min
iJO1366	1805	2583	1687	1242	192.22 min	38.43 min	21.93 min	18177.00 min
Recon1	2766	3741	2467	1414100	308.97 min	51.71 min	36.20 min	22268.00 min

In both ACHR and CHRR the number of initial iterations that have been discarded at the beginning of the sampling (warm up) was set to *M*_*warm*_ = 20, 000. The design of the OPTGP algorithm is such that it always generates a fixed number (2*n*) of warm up points. For BMFA there is no warm up phase since its Gibbs algorithm starts out from the posteriori mode of a truncated normal distribution.

In the BMFA framework the variances *γ*_*i*_ around the relaxed steady state condition of Eq (11) were set to *γ*_*i*_ = 0.0001 (*i* = 1…*m*), as in Heinonen et al. [22]. Defining a nearly strict steady state condition by using such small variances (*γ*_*i*_) should not have a large impact on generated samples by Gibbs algorithm. The average flux ranges (AFR) reported in Table 2 indicate that the models have different flux ranges and in the BMFA framework, the prior variances for fluxes should be adjusted according to the flux ranges. For all models except iLJ478 and iSB619, the prior variances for fluxes were set to (*min*(0.5(**v**^*ub*^ − **v**^*lb*^), 1000))^2^ to cover the flux ranges. To avoid numerical instabilities in the covariance matrix for the iLJ478 and iSB619, the prior variances were set to (*min*(0.5(**v**^*ub*^ − **v**^*lb*^), 500))^2^ and (*min*(0.5(**v**^*ub*^ − **v**^*lb*^), 100))^2^, respectively.

The implementations of the ACHR and CHRR algorithms available via the *sampleCbModel()* function from the COBRA toolbox (version 3.0) [18] of Matlab was used. The *bmfa()* from the COBRA toolbox was applied to generate the samples based on the Gibbs algorithm used in the BMFA. We have made a minor change in the script of the *bmfa()* function in order to allow the user to adjust the prior variations for fluxes according to the flux ranges of a metabolic model. The samples from OPTGP algorithm were drawn using the *optGPSampler()* function from the COBRA toolbox in Python (COBRApy) [34].

Three of the algorithms (ACHR, OPTGP and CHRR) were run on a computer with an Intel Core i7 processor (2.5 GHz). The run time of the algorithms while sampling each of the ten models were measured using *tic/toc* function in Matlab and *time* function in Python which reports the elapsed “wall-clock” time (Table 2). Both OPTGP and CHRR were run in parallel on four threads, while both ACHR and Gibbs were run on a single thread, since their current implementation can not exploit parallelism. The more computationally demanding Gibbs algorithm was run on a server with 32 Cores (2.7 GHz). A pro-rata conversion was applied in order for its run time to be comparable to that of the three other algorithms. To this end 200 samples from the Gibbs sampler were generated on the Intel Core i7 processor, and the corresponding run time formed the basis of the conversion factor.

## Convergence diagnostics

The *M* = 20, 000 samples from each algorithm have been validated and compared in R [29]. A sample generated by a MCMC algorithm is guaranteed to be representative of the true flux distribution only if the sample chain has converged (in distribution). It is hence customary to apply one or more convergence diagnostics to avoid incorrect inference [6]. In the present study we investigated and compared four different convergence diagnostics. Distributional convergence may be assessed within a chain or across multiple chains run in parallel, started from different values inside the solution space. Not all the implementations of the algorithms used here allows the starting to be controlled, so we focused our comparison on single-chain diagnostics. The diagnostics were applied separately to each flux of a model, and we have presented the proportion of converged chains as a summary statistic.

When applying a MCMC method there are three constants that must be specified. First, the number of warm up samples, *M*_*warm*_, determines how many samples must be discarded initially before distributional convergence is achieved. Then sampling continues for *M* iterations, which yields the sample *v*^(1)^, …, *v*^(*M*)^ that is used for inference. The third constant is the so called “thinning” parameter, which in the current study was set to 1000 in all sampling algorithms. This means that only every 1000th sample from the underlying Markov chain was kept. The purpose is to reduce the autocorrelation. Note that autocorrelation in the chain per se does not invalidate the inference drawn, but it reduces the information content.

Below the four diagnostic tools are reviewed briefly. For more details the reader is referred to the background papers. We let *v*^(1)^, …, *v*^(*M*)^ denote the sample chain for one specific flux.

### Raftery and Lewis

Based on a single chain of flux samples (pilot chain), *v*^(1)^, …, *v*^(*M*)^, the Raftery and Lewis diagnostic [35] provided an estimate of the number of iterations in the warm up phase, *M*_*warm*_, and the required number of further iterations, *N*_*max*_, to estimate the quantile *q* to within a precision of ±*e* with probability *p*. It further determined the minimum number of iterations, *N*_*min*_, that should be run as a pilot chain assuming independent samples. Using these statistics, this test determined a dependence factor *I* = (*M*_*warm*_ + *N*_*max*_)/*N*_*min*_ as a measure of dependency between consecutive samples (autocorrelation). Here we considered the chains with *I* > 5 as highly autocorrelated chains that were not run long enough. Here, all statistics in Raftery and Lewis diagnostic were calculated to estimate a quantile of 0.025 to within a precision of ±0.005 with probability 0.95 using the *raftery.diag()* function from the *CODA* R package [36].

### Geweke

Geweke [37] proposed a single-chain convergence diagnostic which compares the average value of the first and last segments of the chain *v*^(1)^, …, *v*^(*M*)^. Let *B*_1_ denotes the first 10% of the samples, and *B*_2_ denotes the last 50%. The test statistic for the Geweke diagnostic is the *Z*-score

$$\begin{array}{c} Z=\frac{{\bar{B}}_{1}-{\bar{B}}_{2}}{\sqrt{\sigma_{{\bar{B}}_{1}}^{2}+\sigma_{{\bar{B}}_{2}}^{2}}}, \end{array}$$ (12)

where ${\bar{B}}_{1}$ and ${\bar{B}}_{2}$ are the averages of the two segments, and $\sigma_{{\bar{B}}_{1}}^{2}$ and $\sigma_{{\bar{B}}_{2}}^{2}$ are the associated standard errors. If the chain has converged in distribution, ${\bar{B}}_{1}$ and ${\bar{B}}_{2}$ have the same expected (mean) value. When *M* is sufficiently large, ${\bar{B}}_{1}$ and ${\bar{B}}_{2}$ will approximately be normally distributed, and *Z* will follow a standard normal distribution. Here, the Z-score was computed using the *geweke.diag()* function from the *CODA* package in R [36]. The convergence criterion for the Geweke diagnostic is |*Z*|≤1.28.

### Interval Based Scale Reduction Factor (IPSRF)

Our third convergence diagnostic is based on the Gelman-Rubin diagnostic [38]. This is originally a multiple-chain diagnostic which compares the difference in across- and within-chain variances. The idea is that if all chains have converged the sample variances will be the same. The original Gelman-Rubin diagnostic assumes normality of the samples. As a typical flux distribution is not normal for a genome scale metabolic model [6], a modified version known as the Interval-based potential reduction factor (IPSRF) should instead be used [39].

To apply the IPSRF diagnostic to a single chain, the first and last third of the chain can be treated as two “parallel” chains. The resulting IPSRF value was estimated using the *ipsrf()* function in the MCMC diagnostics toolbox in Matlab. The test criterion is IPSRF < 0.9 or IPSRF > 1.1, in which case the single chain was considered to have not converged.

### Hellinger distance

The Hellinger distance is a density based convergence diagnostic that can be used for a single chain or multiple chains [40]. The basic idea is to compare the flux density estimated from the first third segment of the chain, *p*_1_(*v*), with that of the last third segment, *p*_3_(*v*). The probability densities *p*_1_ and *p*_3_ are calculated using the *densityfun()* function of the *statip* package in R [41]. The Hellinger distance statistic is defined as

$$\begin{array}{c} HD\left(p_{1},p_{3}\right)=\sqrt{\frac{1}{2}\int_{-\infty }^{\infty }{\left(\sqrt{p_{1}\left(v\right)}-\sqrt{p_{3}\left(v\right)}\right)}^{2}dv}. \end{array}$$ (13)

It is a proper metric, symmetric in *p*_1_ and *p*_3_. Further, it is bounded by 0 ≤ *HD* ≤ 1, where 0 indicates no divergence and 1 indicates no common support between the two distributions. As suggested by Boone et al. [40], if the Hellinger distance between the two probability density functions of two segments was less than 0.1 (*HD* ≤ 0.1), then the chain has been considered to have converged else not. We wrote a script in R to calculate the Hellinger distance where we used the *integral()* from the *pracma* package [42].

## Comparison of algorithms

### Correlation coefficient

The two most important statistical summaries of a sample *v*^(1)^, …, *v*^(*M*)^ are its mean and variance:

$$\begin{array}{c} \bar{v}=\frac{1}{M}\sum_{l=1}^{M}v^{\left(l\right)}\;\text{and}\;s^{2}=\frac{1}{M-1}\sum_{l=1}^{M}{\left(v^{\left(l\right)}-\bar{v}\right)}^{2}. \end{array}$$ (14)

If two sampling algorithms yield the same flux distributions, they should give the same values of $\bar{v}$ (and similarly for *s*^2^) for a given reaction. We compare algorithms in terms of their Pearson correlation across reactions for both of these quantities. In term of the sample average the Pearson correlation between Algorithm 1 and 2 is given as

$$\begin{array}{c} r=\frac{\sum_{j=1}^{n}[\left({\bar{v}}_{j,1}-{\bar{\bar{v}}}_{1}\right)\left({\bar{v}}_{j,2}-{\bar{\bar{v}}}_{2}\right)}{\sqrt{\sum_{j=1}^{n}{\left({\bar{v}}_{j,1}-{\bar{\bar{v}}}_{1}\right)}^{2}{\left({\bar{v}}_{j,2}-{\bar{\bar{v}}}_{2}\right)}^{2}}} \end{array}$$ (15)

where ${\bar{v}}_{j,1}$ is the sample average for the *j*th flux, and ${\bar{\bar{v}}}_{1}=\frac{1}{n}\sum_{j=1}^{n}{\bar{v}}_{j,1}$ is the across-flux average, both for Algorithm 1 (and similar quantities for Algorithm 2). The Pearson correlation is well suited as a measure of association because the flux average $\bar{v}$ will be approximately normally distributed by the central limit theorem. Further, *r* varies between −1 and +1. A perfect positive (linear) association is indicated by a value of + 1, while 0 represents no association [43].

We used CHRR as a reference in the comparison with the three other algorithms. Outliers were determined in the following way, and subsequently omitted when calculating the Pearson correlation. In the case of CHRR versus ACHR, say, a reaction was considered an outlier if the difference ${\bar{v}}^{\text{CHRR}}-{\bar{v}}^{\text{ACHR}}$ exceeded 2 standard deviations (of this difference, across reactions). A similar outlier criterion, based on *s*^CHRR^ − *s*^ACHR^, was applied on the sample standard deviations *s*. The set of omitted reactions includes the outliers in both the means and the standard deviations of the flux values. The value of the Pearson correlation, *r*, is calculated using the *cor()* function from the *stats* package in R [29].

### Kullback-Leibler divergence

We also compared the distributional shape resulting from different algorithms, using the Kullback-Leibler divergence (KLD) as a measure of dissimilarity. Let *p*_1_(*v*) and *p*_2_(*v*) denote flux densities resulting from two algorithms, and define

$$\begin{array}{c} KLD\left(p_{2}|p_{1}\right)=\int_{-\infty }^{\infty }\ln(\frac{p_{1}\left(v\right)}{p_{2}\left(v\right)})p_{1}\left(v\right)dv. \end{array}$$ (16)

It may be shown that *KLD*(*p*_2_|*p*_1_)≥0, and that it is zero only if *p*_1_ and *p*_2_ are identical functions [44]. Note that *KLD*(*p*_2_|*p*_1_) is not symmetric in *p*_1_ and *p*_2_, we will refer to *p*_1_ as the *reference*. The CHRR will be used as the reference against the three other methods. A script has been written in R to calculate the KLD in Eq (16). The probability densities *p*_1_ and *p*_3_ are calculated using the *density()* function of the *stats* package in R [29].

We classified the accuracy of the approximation as *good* agreement *KLD* < 0.05, *medium* agreement 0.05 ≤ *KLD* ≤ 0.5 and *poor* match *KLD* > 0.5. This classification was adopted from De Martino et al. [17].

### Effective sample size

The effective sample size (ESS) of an autocorrelated MCMC sample of size *M* is the equivalent number of independent draws from the target distribution. Gelman et al. [45] defines the effective sample size (for sample mean) as

$$\begin{array}{c} ESS=\frac{M}{1+2\sum_{k=1}^{\infty }\rho_{k}}, \end{array}$$ (17)

where *ρ*_*k*_ is the autocorrelation at lag *k*. From a given sample *v*^(1)^, …, *v*^(*M*)^ the estimate of *ρ*_*k*_ is given as

$$\begin{array}{c} \rho_{k}=\frac{\frac{1}{M-k}\sum_{l=1}^{M-k}\left(v^{\left(l\right)}-\bar{v}\right)\left(v^{\left(l+k\right)}-\bar{v}\right)}{\frac{1}{M}\sum_{l=1}^{M}{\left(v^{\left(l\right)}-\bar{v}\right)}^{2}}, \end{array}$$ (18)

where $\bar{v}$ is the mean of the samples [46]. Due to the random walk like behaviour of MCMC algorithms, one typically has 0 ≤ *ρ*_*k*_ ≤ 1 which implies *ESS* ≤ *M*. A low value of ESS/M indicates that the algorithm generates highly autocorrelated samples (large *ρ*_*k*_). The higher the autocorrelation is, the less information about the target distribution is contained in a sample of fixed size. Increasing the value of the thinning parameter will reduce the autocorrelation, but this gain comes at a computational cost.

In order to compare the efficiency of two algorithms in terms of ESS, the computation time must be taken into account since one algorithm may generate a larger number of independent samples slowly, while another may generate highly autocorrelated samples fast. The efficiency of each algorithm in generating independent samples per time unit for each individual flux was measured by

$$\begin{array}{c} E=\frac{ESS}{Run\;time}, \end{array}$$ (19)

where the ESS value has been calculated with the *effectiveSize()* function from the *CODA* R package [36] and the run time is reported in Table 2 for each algorithm across the ten models.

## Results

The sampling algorithms have been compared on the ten metabolic models using the criteria described earlier. First, the degree of convergence was investigated. Secondly, the flux densities generated by the different algorithms were compared. Finally, the computational efficiency of the algorithms was assessed.

We were only able to successfully apply the *xsample()* algorithm in one (E. coli core) out of the ten models (details given below). Hence, the comparison of algorithms was performed only between CHRR, ACHR, OPTGP and the Gibbs sampler.

### Convergence of algorithms

**Fig 2:**
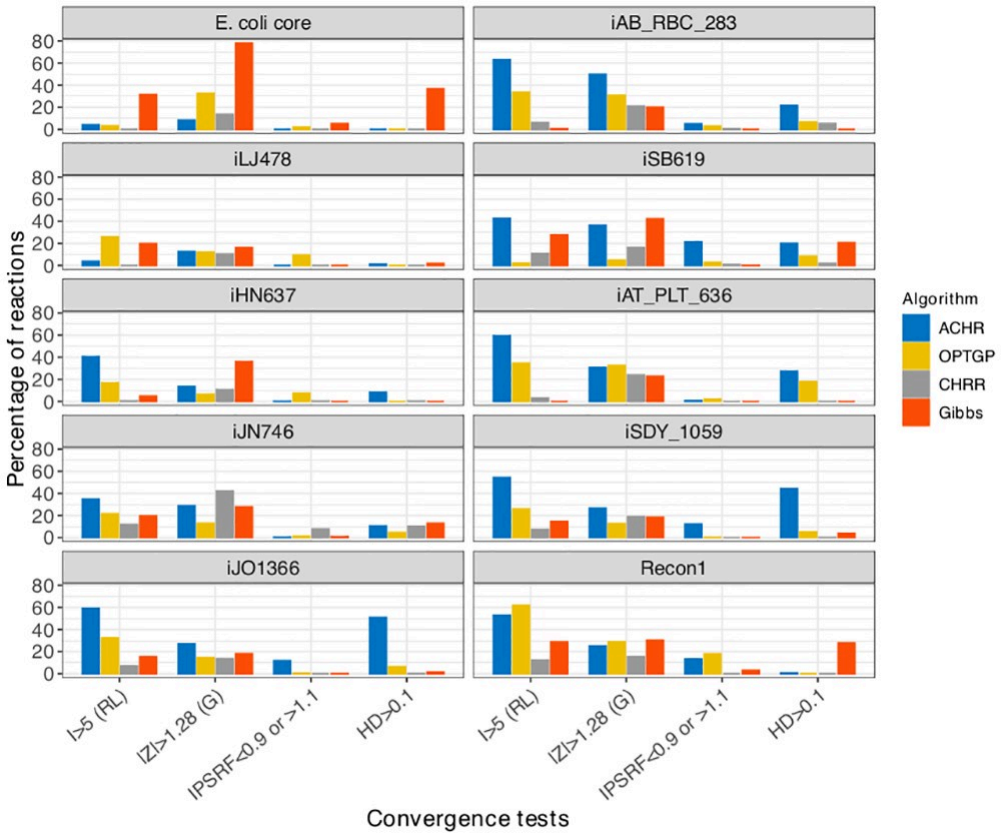
Four convergence diagnostics across four algorithms and ten models. The vertical axis shows the proportions of reactions in each model rejected by the different convergence tests: Raftery and Lewis (RL), Geweke (G), IPSRF and Hellinger distance (HD) on the horizontal axis.

For all ten models, the convergence of the generated samples was assessed (by reaction) via the four single-chain convergence diagnostics. Fig 2 shows the percentage of reactions that failed for each of the Raftery and Lewis diagnostic (*I* > 5), Geweke test (∣*Z*∣>1.28), IPSRF test (IPSRF<0.9 or IPSRF>1.1) and Hellinger distance test (*HD* > 0.1). In the majority of the models, CHRR was the algorithm with the least convergence problems. All four diagnostics agree on this, but when it comes to the ranking of ACHR, OPTGP and Gibbs sampler, the diagnostics tell less coherent stories, so it is difficult draw general conclusions. ACHR did however seem to have convergence problems for many models, and the Gibbs sampler had problems for E. coli core in particular.

We only show summaries statistics for the diagnostics. It was also possible to inspect convergence for individual reactions, and when doing so we found that it is not necessarily the same reactions that failed to converge according to the different diagnostics. Therefore a combination of convergence diagnostics should be used to make a certain decision about sampling convergence. Apparently, the IPSRF test is more liberal in accepting convergence, but it should be noted that this conclusion is specific to our chosen settings (the default) for that diagnostic.

### Comparison means and standard deviations

**Fig 3:**
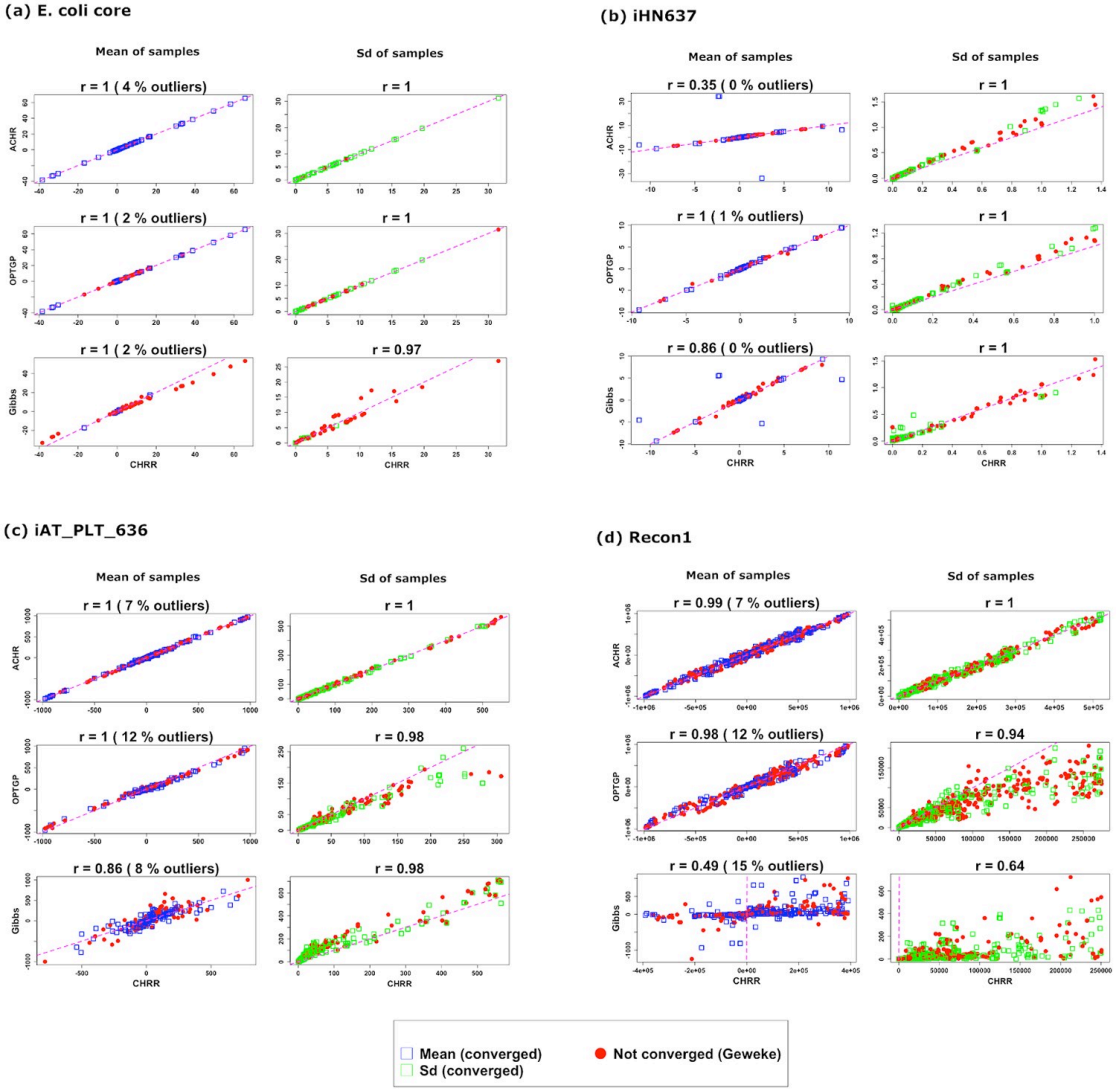
Scatter plot of sample means (blue) and standard deviations *s* (green) for ACHR, OPTGP and the Gibbs sampler (vertical axis) versus CHRR (horizontal axis) for four models. Sample means ($\bar{v}$) and standard deviations (*s*) are calculated according to Eq (14). The Pearson correlation *r* is shown on top of each scatter plot, and the proportion of outliers removed is given in parenthesis. The sample means and standard deviations marked in red correspond to the reactions for which at least one of the two algorithms in a comparison failed the Geweke test. The identity line (pink dashed) is included to ease comparison.

Fig 3 compares CHRR against each of three other algorithms in terms of sample means ($\bar{v}$) and standard deviations (*s*) as given by Eq (14). The figure only shows four models (E. coli core, iHN637, iAT_PLT_636 and Recon1), but plots for the remaining six models are provided in the online Supplementary (S1 Fig).

In general, the four algorithms returned very similar sample means $\bar{v}$, as can be seen from the fact that the points in the plot lie along the identity line. This is also reflected in a Pearson correlation close to *r* = 1. The exception is the Gibbs sampler (versus CHRR), especially for the Recon1 model. For this model the range of $\bar{v}$ values was much smaller for the Gibbs sampler than for CHRR. Note, however, that the Pearson correlation is substantial (*r* = 0.50), which implies that there is still a strong linear relationship, although with slope different from 1. The same effect, but to a much smaller degree, is also observed for the iAT_PLT_636 model. The effect is known as “shrinkage-toward-zero”, and is caused by the prior distribution applied to fluxes in the Gibbs algorithm. Ideally, such priors should be made “non-informative” by choosing the prior variance sufficiently large, but in the case of Recon1 it was not possible to make the prior cover the full flux range (AFR in Table 2) without encountering numerical problems in the Gibbs sampler.

Fig 3 includes also the reactions for which the algorithms did not converge, but reactions for which at least one of the two algorithms in a comparison failed the Geweke test are marked in red. For E. coli core there is a tendency that the largest fluxes (negative or positive) face convergence problems for the Gibbs sampler, while for the other algorithms and models there is no such clear pattern. Recall that Fig 2 summarized convergence for each algorithm separately.

The standard deviations from ACHR, OPTGP and CHRR agree well in general, i.e their green points lie close to the identity line. For the Recon1 model, OPTGP has lower variance than CHRR, and there is more spread (*r* = 0.94). The Gibbs sampler is in fairly good agreement with CHRR, but for Recon1 its standard deviations are much smaller than those from the Gibbs sampler. This reflects the shrinkage-toward-zero effect caused by the narrow Bayesian priors applied in the Gibbs sampler, as discussed above. For iAT_PLT_636 the standard deviations from the Gibbs sampler exceed those of CHRR, indicating that the Gibbs sampler is better (than CHRR) able to explore the flux space for this model.

The % outliers shown on top of each plot indicates the percentage of reactions for which large differences have been observed between the sample means or standard deviations from two algorithms. Note that in the plots of standard deviations the reactions with the standard deviations smaller than 99% quantile have been included.

### Comparison of marginal distributions

**Fig 4:**
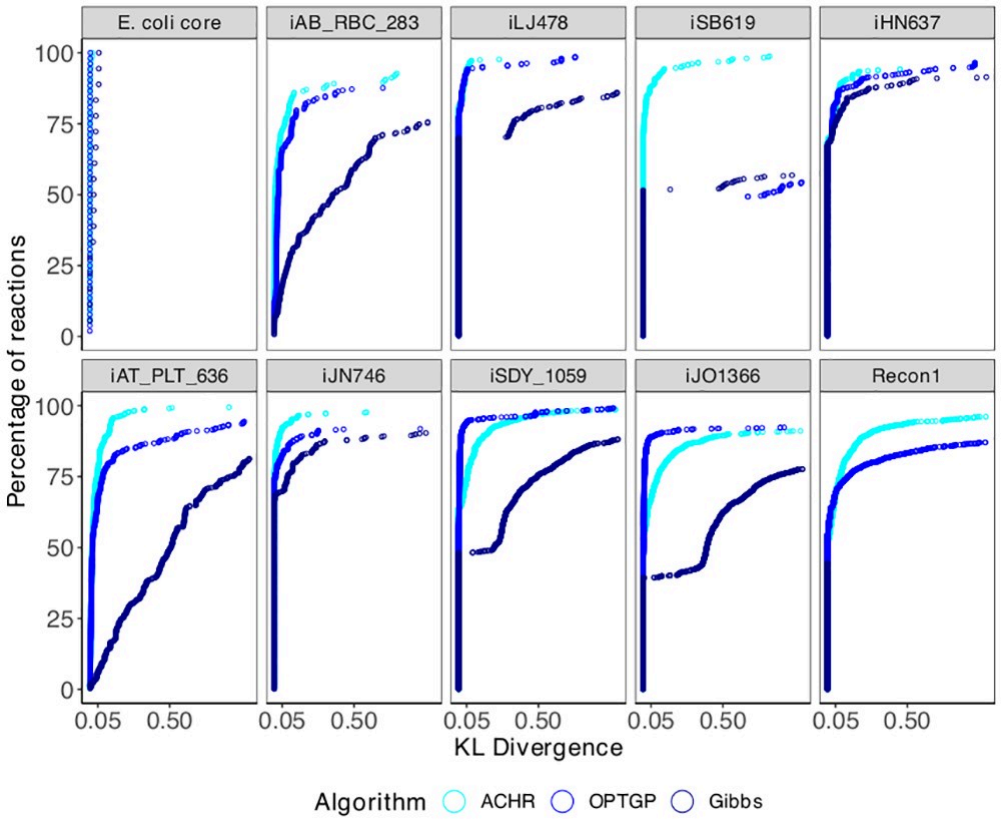
Comparison of flux densities between algorithms by model in terms of the KL divergence. Each plot shows the cumulative distribution functions of KLD across reactions, as defined in Eq (16) with CHRR as the reference.

While Fig 3 compares algorithms only in terms of sample mean and standard deviation, Fig 4 compares the full distributional shape of the flux densities. The figure shows cumulative distribution function of KLD (Kullback-Leibler divergence) across reactions, where CHRR is used as the reference for each of ACHR, OPTGP and the Gibbs sampler. Only reactions for which both algorithms in a comparison, ACHR and CHRR say, converged according to the Geweke diagnostic are included in the figure. The KLD is affected by discrepancies in means and standard deviations, so any off-diagonal reactions in Fig 3 will result in a large KLD value. In addition, Fig 4 shows differences caused by different degree of skewness in the densities.

**Fig 5:**
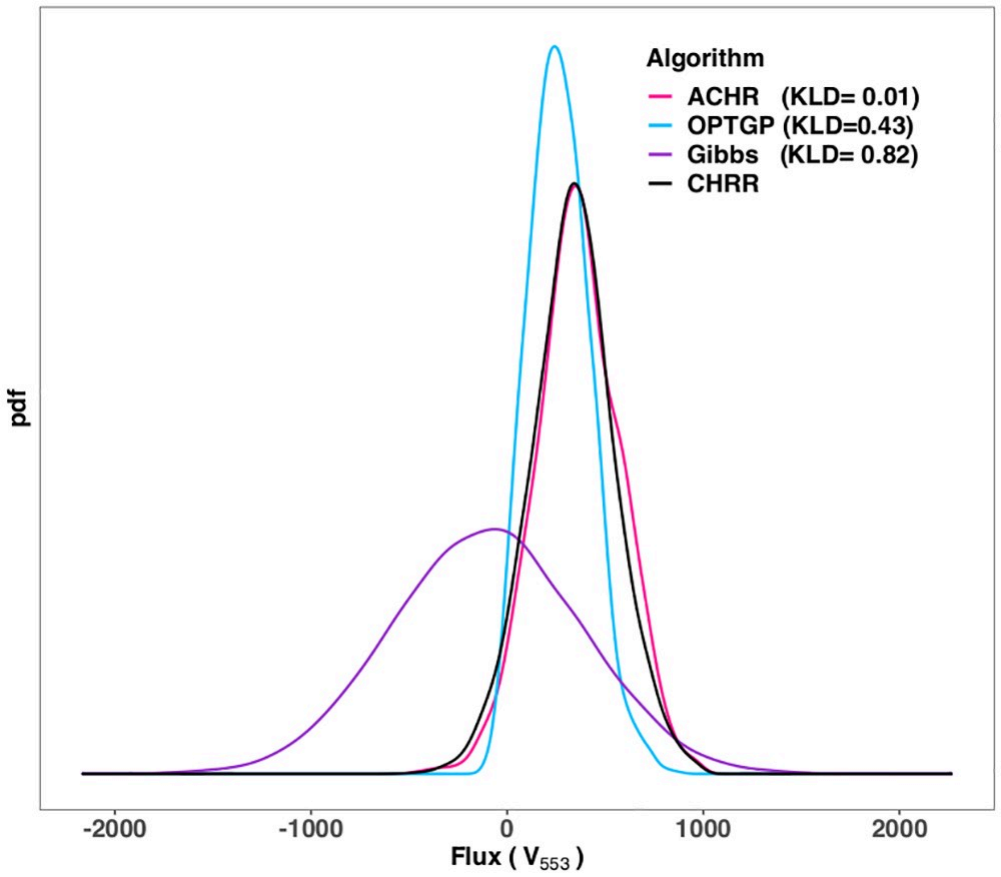
Flux densities resulting from different algorithms and corresponding KLD values (relative to CHRR). The reaction shown is the Fumarase mitochondrial reaction (*v*_553_) of the iAT_PLT_636 model.

Before discussing the results in Fig 4, we recall the qualitative (*good*–*medium*–*poor*) scale of the KL divergence (KLD). To get a visual impression of what this amounts to in a density plot, Fig 5 shows flux densities and KLD values for the Fumarase mitochondrial reaction (*v*_553_) of the iAT_PLT_636 model. According to this KLD scale ACHR has a good similarity to CHRR (*KLD* = 0.01 < 0.05), and OPTGP has a medium similarity to CHRR (0.05 < *KLD* = 0.43 < 0.5) while the Gibbs algorithm has a poor similarity to CHRR (*KLD* = 0.82 > 0.5). Returning to Fig 4, it is seen that almost all of the reactions of the iHN637 model are in the *good* category for all three algorithms. The E. coli core model is the only model for which both the ACHR, OPTGP and the Gibbs algorithm present good consistency with CHRR for all reactions (*KLD* < 0.05). For the other eight model, however, a large proportion of the reactions are in the *poor* category. Taking iAB_RBC_283 as an example, for the Gibbs sampler approximately 50% of the reactions have *KLD* > 0.5. For ACHR and OPTGP the proportion with *KLD* > 0.5 is somewhat lower (10-15%). In Recon1 a large proportion of the reactions are outside the range of the horizontal axis for the Gibbs sampler, and hence do not show in the plot. These reactions are affected by the shrinkage-towards-zero effect displayed in Fig 3.

The general message from Fig 4 is that ACHR is producing flux distributions most similar to CHRR. This conclusion is based on the fact that its cumulative distribution curve (cyan) lies above the two others. The latter does not preclude ACHR having a lower *KLD* value than OPTGP, say, for individual reactions, but it is a statement that is valid as a summary across all reactions. For the majority of the ten models, OPTGP was much closer to ACHR in comparison to the Gibbs sampler. The only exception to this was the iSB619 model for which the cumulative distribution function for OPTGP lies below that of Gibbs sampler. In conclusion, ACHR has the highest consistency with CHRR, followed by OPTGP. The Gibbs sampler is ranked as the least consistent method with CHRR. The latter is most likely due to the shrinkage-towards-zero effect caused by the use of informative priors in the Gibbs sampler.

### Sampling efficiency

**Fig 6:**
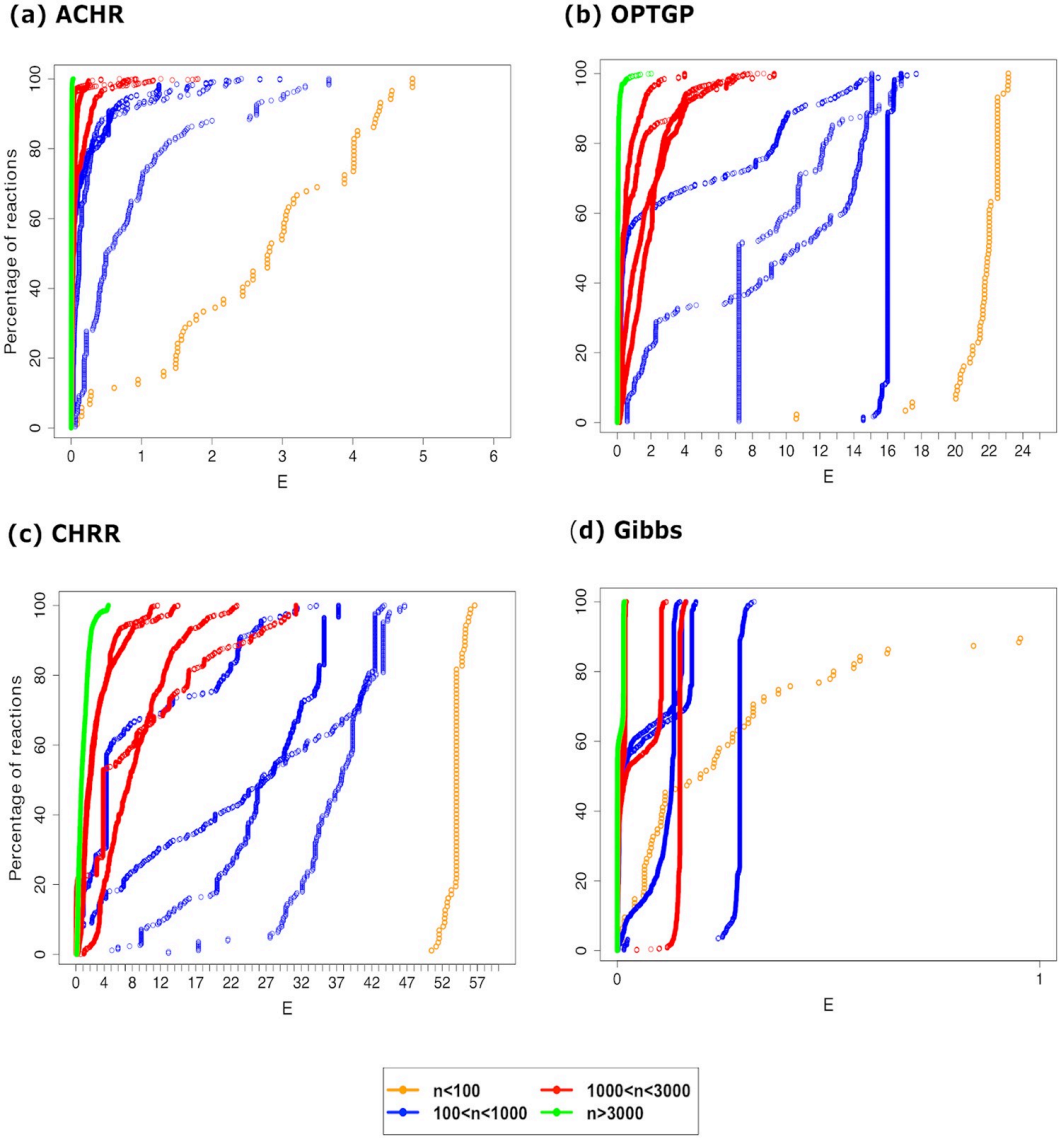
Comparison of sampling efficiency across four algorithms and ten models. The vertical axis shows the proportions of reactions being less than a given value of the efficiency measure *E* on the horizontal axis. The ten different curves correspond to the ten models which are classified in four groups according to their number of reactions (see legend).

Fig 6 compares the cumulative distribution functions for the efficiency measure *E*, given by Eq (19), of the different metabolic models, separately for each sampler. Recall that for two curves that never cross each other, such as the yellow (E. coli core) and any of the blue curves in Panel a), the distribution of *E* for one model (blue) is stochastically larger than the other (yellow).

The models have been categorized in four groups based on the number of reactions: *n* < 100, 100 < *n* < 1000, 1000 < *n* < 3000 and *n* > 3000. The yellow curve (E. coli core) has the highest effective sample size per time unit for all four algorithms. This was expected as E. coli core is the smallest model (*n* = 95 reactions). If it can be assumed that the number of metabolites (*m*) is proportional to *n*, the computation time for the matrix vector product *S* **v** in Eq (2) grows as *n*^2^ (ignoring that *S* is a sparse matrix). Assuming that the product *S* **v** constitutes the main computational task of any of the sampling algorithm, we expect *E* will decrease proportionally to *n*^−2^ as *n* increases. This theoretical expectation is confirmed, at least qualitatively, in Fig 6 for all four sampling algorithms. The largest model, Recon1, has very low sampling efficiency.

When comparing the four algorithms, we first note that the scales on the horizontal axes differ across panels in Fig 6. The CHRR has the highest sampling efficiency, followed by the ACHR, then by the OPTGP, and finally by the Gibbs sampler. Note that ACHR and CHRR sample the reduced models (of size *n*_*red*_), while OPTGP and Gibbs sample the full models (of size *n*). We see from Table 2 that *n*/*n*_*red*_ is never larger than 2, and attempting to account for model size by multiplying the efficiency of the Gibbs sampler by 4, it is observed that the Gibbs algorithm is still the algorithm with least efficiency.

**Fig 7:**
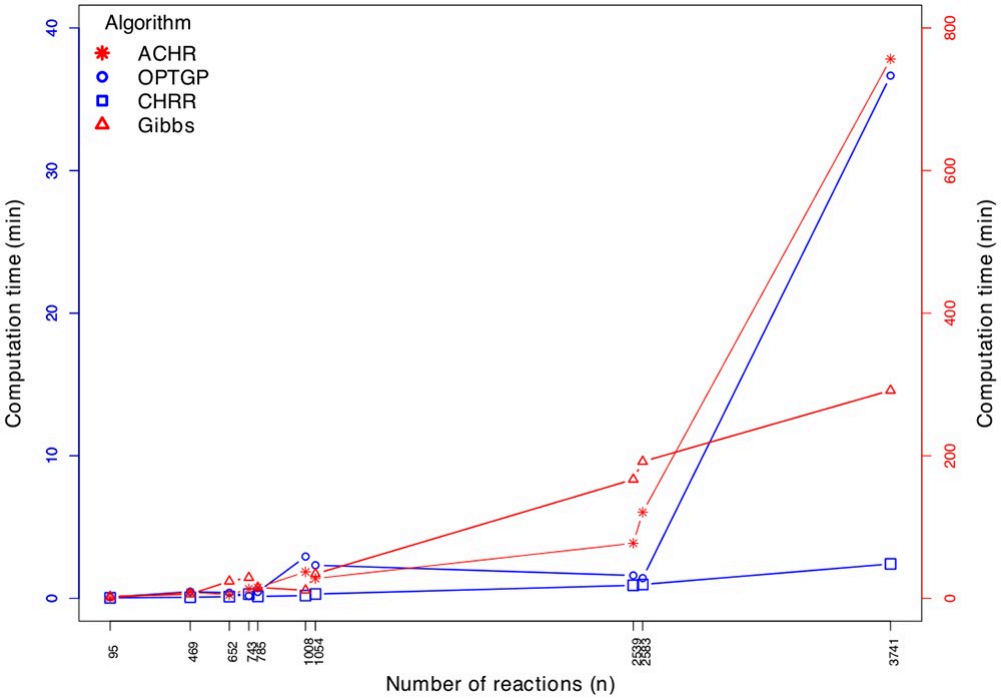
Computation time needed to generate 100 uncorrelated samples by model size (*n*) and algorithm. Each value of *n* shown on the horizontal axis correspond to one of the ten metabolic models, and is taken from Table 2. The left vertical axis is used for OPTGP and CHRR, while the right vertical axis belongs to ACHR and the Gibbs sampler.

To further illustrate how sampling efficiency depends on model size we computed the time it takes to generate 100 independent (uncorrelated) samples. This was computed as 100(*mean*(*ESS*))^−1^ * (Run time) = 100(*mean*(*E*))^−1^ where run time is provided in Table 2 and *E* is given by (19), and the results are shown in Fig 7. As expected, the computation time in general increases with model size, but there are exceptions to this (values of *n* in the rage 1000 to 2500 for OPTGP). These exceptions show that there are other aspects of a model than *n* that determines sampling efficiency. For most of the models, ACHR and the Gibbs sampler (right vertical axis) are slower than OPTGP and CHRR (left axis). We observe that ACHR is the slowest algorithm to generate 100 independent samples, closely followed by the Gibbs sampler which we recall performs sampling on the full models.

**Fig 8:**
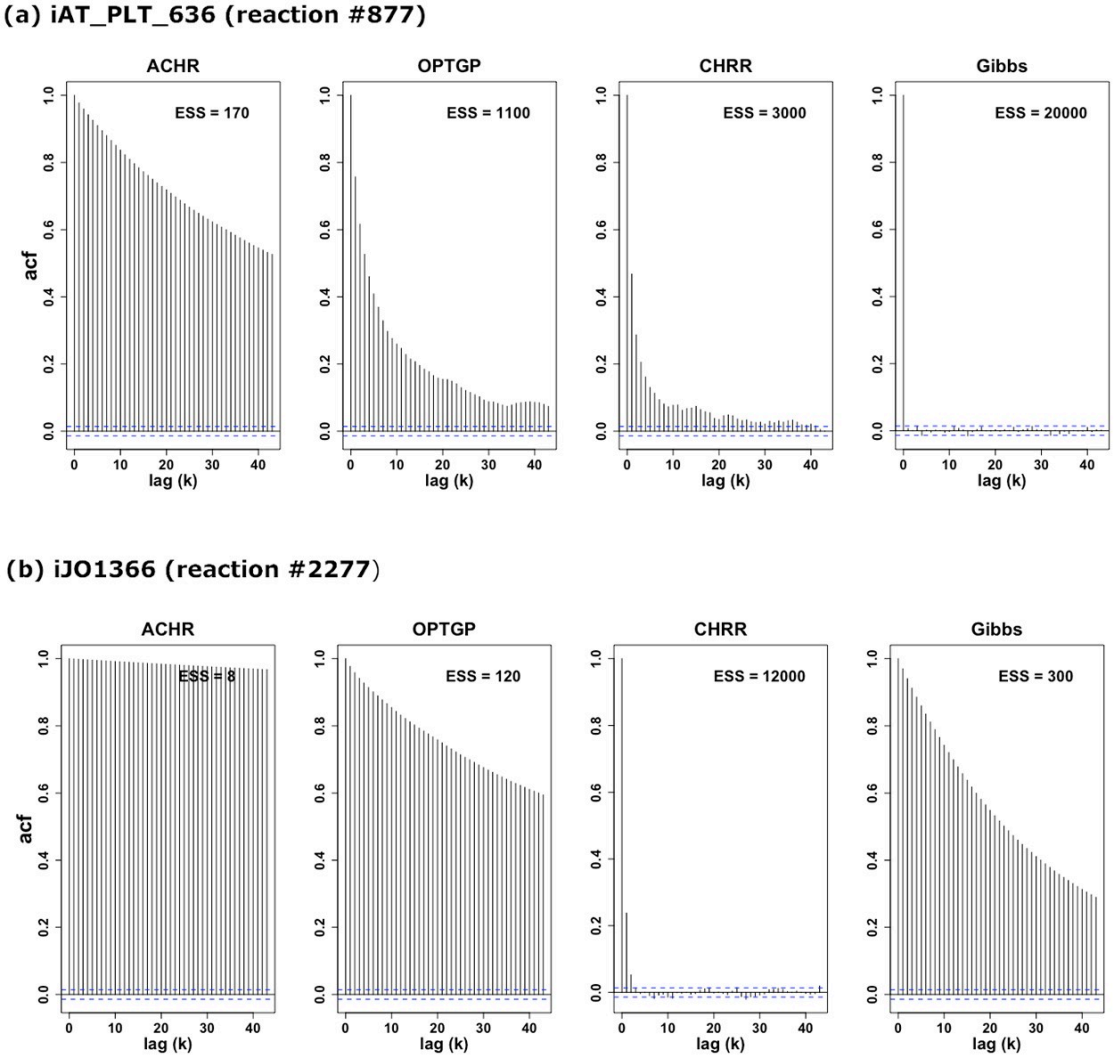
Autocorrelation *ρ*_*k*_ (acf) by lag for the flux *v*_877_ in the iAT_PLT_636 model (Panel a) and the flux *v*_2277_ in the iJO1366 model (Panel b) for each sampling algorithm. These fluxes, *v*_877_ and *v*_2277_, correspond to 1D-myo-Inositol 4-phosphate phosphohydrolase and Ribose-1,5-bisphosphokinase reactions in the iAT_PLT_636 and iJO1366, respectively. The dotted blue lines indicate lag-wise 95% confidence intervals (CIs).

To shed further light on differences in sampling efficiency between algorithms, we inspected the autocorrelation functions *ρ*_*k*_, given by Eq (18), for two individual reactions (Fig 8). Also shown in the figure is the corresponding measure of effective sample size (ESS) defined in Eq (17). The algorithms differ widely in how fast *ρ*_*k*_ decayed as a function of *k*, and consequently, in the value of ESS. We note, however, that the numerical values shown in Fig 8 are specific to the value of the thinning parameter (1000) used, so absolute values are not relevant. The ACHR was the algorithm with the lowest ESS, followed by OPTGP. For the iAT_PLT_636 model (Panel a), the Gibbs sampler yields an almost uncorrelated chain, meaning that the thinning parameter could have been set to a lower number than 1000, as we currently are discarding some useful information about the flux distributions. For iJO1366 (Panel b), CHRR had almost no autocorrelation, while the Gibbs sampler had a substantial autocorrelation. This shows that the details of the model plays an important role in determining which algorithms is the most efficient in terms of generating independent samples.

### Performance of *xsample()*

The *xsample()* function in R [29] was attempted on the reduced versions of the ten metabolic models, but we were only able to successfully run it for the E. coli core model. The reason for the problem may be the large variation in flux ranges for the nine other models. For instance, the minimum and maximum of the flux ranges were of orders 10^−6^ and 10^3^, respectively, in the reduced version of iAB_RBC_283. The jump length is a compromise to sample over these 9 orders of magnitude in which a small jump length is needed for the fluxes with small range and a large jump length is needed for the fluxes with large range. In the *xsample()* function, the jump lengths which are the diagonal elements of the matrix Ω in Eq (10) were set to $0.5(v_{red}^{ub}-v_{red}^{lb})$ in order to scale them to the range of the fluxes. However having large step lengths made the sampling algorithm very inefficient since a lot of mirroring steps were required and the algorithm rejected many draws in each iteration.

We also tried $0.01(v_{red}^{ub}-v_{red}^{lb})$ for the jump lengths, and the algorithm was able to sample all the models, albeit very slow. Checking the generated samples, we observed that since the jump lengths were small the sampler moved barely from the initial flux vector. Due to this the generated samples were highly autocorrelated and we have not included them in the further analysis. So the best choice of jump lengths as a hyper parameter in the *xsample()* was not trivial and one has to use a cluster with simply a lot of brute computing power to deal with this.

For the 20, 000 samples that were successfully obtained from the E. coli core model, applying the jump lengths $0.5(v_{red}^{ub}-v_{red}^{lb})$, a statistical analysis was performed similar to that above for the other algorithms. The rates of non-convergence according to the four diagnostic tests were: 0% (Raftery and Lewis), 18.9% (Geweke), 0% IPSRF, and 0% (Hellinger distance). These are lower than for the other algorithms, except for the Geweke test, but still considerably lower than the Gibbs sampler (Fig 1). However, the run time of the *xsample()* to generate the samples for the E. coli core model was considerably larger than the Gibbs sampler. The scatterplots of sample means and standard deviations against CHRR look qualitatively similar to those for the Gibbs sampler in Fig 3.

## Discussion and conclusion

In this study we have reviewed and compared five MC sampling algorithms for constraint-based modeling of metabolic networks (Table 1). The algorithms have been classified as allowing either a deterministic and stochastic formulation of the metabolic model (Fig 1). In the stochastic formulation, which is the most general, the steady state assumption can be relaxed and noisy flux observations can be incorporated in the model. However, to ensure a fair comparison of algorithms, all experiments were done considering no flux measurements.

We have reviewed and compared four standard convergence diagnostics that can be used to check if the algorithms have been run for sufficiently many iterations that the samples come from the target flux distribution. Finally, important metrics for comparing the algorithms have been similarity of flux distributions and computational efficiency.

The algorithms have been applied to ten genome scale metabolic networks (Table 2). However, in case of the *xsample()* algorithm we were only able to successfully apply it to a single model (E.coli), so our comparison was done with only four algorithms (ACHR, OPTGP, CHRR and the Gibbs sampler). An efficient sampling algorithm which allows the stochastic framework of Van den Meersche et al. [20] to be applied at genome scale is thus lacking.

Comparing the algorithms in terms of convergence, the CHRR has the least convergence problems. This result is in agreement with the findings in Herrmann et al. [6] in which three algorithms ACHR, OPTGP and CHRR are compared in terms of convergence and run time. We have found that the set of reactions which fail the convergence criterion is not necessarily the same across different diagnostic tests. Also, the proportion of reactions for which a test fails can be substantial (Fig 2). Hence, from a practical perspective it does not seem feasible to require that all reactions have converged before the output from an algorithm can be trusted. Instead, the focus should be on reactions of special interest, and for those reactions one can follow the recommendation of Herrmann et al. [6] that the whole suite of diagnostics should be satisfied. Further, in our comparison of algorithms in Fig 3, there seems to be good agreement between algorithms also for reactions that have not converged.

Convergence to the target distribution is not guaranteed for ACHR and OPTGP, while for CHRR convergence is guaranteed due to its Markovian nature. For this the other algorithms were compared against CHRR. We found that ACHR generates the most similar (marginal) flux distributions to that of CHRR, followed by OPTGP. The Gibbs sampler deviated most from CHRR, which probably is due to the informative prior distribution imposed on some of the models.

When comparing the algorithms in terms of computational efficiency, we found that the CHRR method outperforms the three other algorithms by generating the highest number of independent samples per time unit for each flux. The main parameter that characterize a model is the number of reactions (*n*), but we have also observed that there are other aspects of a model that affect the performance of an algorithm.

Hamiltonian Monte Carlo (HMC) [47] is another sampling technique for exploring the posterior distribution in the Bayesian framework. In Heinonen et al. [22], the HMC was reported to be inefficient compared to Gibbs sampler in the genome scales metabolic models. We tried to apply HMC via the Template Model Builder (*TMB*) package [48] which is a statistical software platform in R [29]. Using the interval based scale reduction factor (IPSRF) [39] as the convergence criterion, we did not get reliable convergence. Most likely, the feasible truncated density region for high dimension models (*n* > 1000) was extremely narrow causing the HMC constantly to hit the boundaries of the polytope.

Our study ranks the CHRR as the best sampling algorithm for cases such as Fig 1b and 1c in which the steady state assumption has to be satisfied strictly and uncertainties in the observed flux values (if there are any) are negligible. The CHRR is currently available in Matlab. If an open-source programming language is preferred, a good alternative is the OPTGP, which is available in Python. For the stochastic formulation, such as Fig 1e, in which the flux observation and their uncertainty are encoded in a model compatible with relaxed steady state assumption, the only sampling algorithm applicable at the genome scale is the Gibbs sampler which is currently available in Matlab. However, this algorithm performs poorly in terms of sampling efficiency.

## Supporting information

S1 FigScatter plot of sample means and standard deviations.The plots are for ACHR, OPTGP and the Gibbs sampler (vertical axis) versus CHRR (horizontal axis) for six models. Sample means ($\bar{v}$) (blue) and standard deviations (*s*) (green) are calculated according to the formulas in the manuscript. The Pearson correlation *r* is shown on top of each scatter plot, and the proportion of outliers removed is given in parenthesis. The sample means and standard deviations marked in red correspond to the reactions for which at least one of the two algorithms in a comparison failed the Geweke test. The identity line (pink dashed) is included to ease comparison.(PDF)

## References

[1] Nelson DL, Cox MM. Lehninger principles of biochemistry. New York: WH Freeman, 2009.

[2] Palsson BØ. Systems biology. Cambridge University Press, 2015.

[3] Strogatz SH. Nonlinear dynamics and chaos: with applications to physics, biology, chemistry, and engineering. CRC Press, 2018.

[4] Orth JD, Thiele I, Palsson BØ. What is flux balance analysis?. Nature biotechnology, 2010, 28(3):245–248. 10.1038/nbt.1614 PMC3108565 20212490

[5] Grünbaum B, Shephard GC. Convex polytopes. Bulletin of the London Mathematical Society, 1969, 1(3):257–300

[6] Herrmann HA, Dyson BC, Vass L, Johnson GN, Schwartz JM. Flux sampling is a powerful tool to study metabolism under changing environmental conditions. NPJ systems biology and applications, 5(1):1–8. 31482008 10.1038/s41540-019-0109-0 PMC6718391

[7] MacGillivray M, Ko A, Gruber E, Sawyer M, Almaas E, Holder A. Robust analysis of fluxes in genome–scale metabolic pathways. Nature Publishing Group, Scientific reports, 2017, 7(1):1–20. 10.1038/s41598-017-00170-3 PMC5427939 28325918

[8] Pakula TM, Nygren H, Barth D, Heinonen M, Castillo S, Penttilä M, Arvas M. Genome wide analysis of protein production load in Trichoderma reesei. Biotechnology for biofuels, 2016,9(1):132 10.1186/s13068-016-0547-5 27354857 PMC4924338

[9] Dyer ME, Frieze AM. On the complexity of computing the volume of a polyhedron. SIAM Journal on Computing, 1988, 17(5):967–974. 10.1137/0217060

[10] Krauth W. Introduction to monte carlo algorithms In Advances in Computer Simulation, Springer, 1989, 1–35

[11] Wiback SJ, Famili I, Greenberg HJ, Palsson BØ. Monte Carlo sampling can be used to determine the size and shape of the steady-state flux space. Journal of theoretical biology, 2004, 228(4):437–447. 10.1016/j.jtbi.2004.02.006 15178193

[12] Bélisle CJ, Romeijn HE, Smith RL. Hit-and-run algorithms for generating multivariate distributions. Mathematics of Operations Research, 1993, (2):255–266.

[13] Turchin VF. On the computation of multidimensional integrals by the Monte–Carlo method. Theory of Probability and Its Applications, 1971, 16(4):720–724. 10.1137/1116083

[14] Almaas E, Kovacs B, Vicsek T, Oltvai ZN, Barabási AL. Global organization of metabolic fluxes in the bacterium Escherichia coli. Nature, 2004, 427(6977):839 10.1038/nature02289 14985762

[15] Kaufman DE, Smith RL. Direction choice for accelerated convergence in hit-and-run sampling. Operations Research, 1998, 46(1):84–95. 10.1287/opre.46.1.84

[16] Megchelenbrink W, Huynen M, Marchiori E. optGpSampler: an improved tool for uniformly sampling the solution-space of genome-scale metabolic networks. PloS ONE, 2014, 9(2). 10.1371/journal.pone.0086587 24551039 PMC3925089

[17] De Martino D, Mori M, Parisi V. Uniform sampling of steady states in metabolic networks: heterogeneous scales and rounding. PloS one, 2015, 10(4). 10.1371/journal.pone.0122670 PMC4388631 25849140

[18] Becker SA, Feist AM, Mo ML, Hannum G, Palsson BØ, Herrgard MJ. Quantitative prediction of cellular metabolism with constraint-based models: the COBRA Toolbox. Nature protocols, 2007, 2(3):727 10.1038/nprot.2007.99 17406635

[19] Haraldsdóttir HS, Cousins B, Thiele I, Fleming RM, Vempala S. CHRR: coordinate hit-and-run with rounding for uniform sampling of constraint-based models. Bioinformatics, 2017, 33(11):1741–1743. 10.1093/bioinformatics/btx052 28158334 PMC5447232

[20] Van den Meersche K, Soetaert K, Van Oevelen D. xsample (): an R function for sampling linear inverse problems. Journal of Statistical Software, 2009, 30 (Code Snippet 1).

[21] Soetaert K, Van den Meersche K, van Oevelen D. limSolve: Solving linear inverse models. Journal of Statistical Software, Code Snippets, 2009, 30(1).

[22] Heinonen M, Osmala M, Mannerström H, Wallenius J, Kaski S, Rousu J, et al Bayesian metabolic flux analysis reveals intracellular flux couplings. Bioinformatics, 2019, 35(14), pp. i548–i557. 10.1093/bioinformatics/btz315 31510676 PMC6612884

[23] Altmann Y, McLaughlin S, Dobigeon N. Sampling from a multivariate Gaussian distribution truncated on a simplex: a review. IEEE, 2014, Workshop on Statistical Signal Processing (SSP),113–116.

[24] Gilks WR, Richardson S, Spiegelhalter DJ. Introducing markov chain monte carlo. Markov chain Monte Carlo in practice, 1996, 1:19.

[25] Lovász L. Hit-and-run mixes fast. Mathematical Programming, Springer,1999, 86(3):443–461. 10.1007/s101070050099

[26] Zhang Y, Gao L. On numerical solution of the maximum volume ellipsoid problem. SIAM Journal on Optimization, 2003, 14(1):53–76. 10.1137/S1052623401397230

[27] Telgen J. Private communication with A. Boneh. 1980.

[28] Berbee HC, Boender CG, Ran AR, Scheffer CL, Smith RL, Telgen J. Hit-and-run algorithms for the identification of nonredundant linear inequalities. Mathematical Programming, 1987, 37(2):184–207. 10.1007/BF02591694

[29] R Core Team. R: A Language and Environment for Statistical Computing. R Foundation for Statistical Computing, Vienna, Austria, 2013. http://www.R-project.org/.

[30] Roberts GO. Markov chain concepts related to sampling algorithms. Markov chain Monte Carlo in practice, 1996, 57:45–58.

[31] Geman S, Geman D. Stochastic relaxation, Gibbs distributions, and the Bayesian restoration of images. IEEE Transactions on pattern analysis and machine intelligence, 1984, (6):721–741. 22499653 10.1109/tpami.1984.4767596

[32] King ZA, Lu J, Dräger A, Miller P, Federowicz S, Lerman JA, Ebrahim A, Palsson BØ, Lewis NE. BiGG Models: A platform for integrating, standardizing and sharing genome-scale models. Nucleic acids research, 2015, 44(D1):D515–D522. 26476456 10.1093/nar/gkv1049 PMC4702785

[33] Mahadevan R, Schilling CH. The effects of alternate optimal solutions in constraint-based genome-scale metabolic models. Elsevier, Metabolic engineering, 2003, 5(4):264–276. 10.1016/j.ymben.2003.09.002 14642354

[34] Ebrahim A, Lerman JA, Palsson BØ, Hyduke DR. COBRApy: constraints-based reconstruction and analysis for python. BMC systems biology, 2013, 7(1):74 10.1186/1752-0509-7-74 23927696 PMC3751080

[35] Raftery AE, Lewis S. How many iterations in the Gibbs sampler? Bernardo J. M., Berger J., Dawid A. P., Smith A. F. M. 4th edn, Oxford: Bayesian Statistics, 1992.

[36] Plummer M, Best N, Cowles K, Vines K. CODA: convergence diagnosis and output analysis for MCMC. R news, 2006;6(1):7–11.

[37] Geweke J. Evaluating the accuracy of sampling-based approaches to the calculation of posterior moments. Oxford: J. O. Berger, A. P. Dawid, Smith A. F. M. (ed. 4) Bayesian Statistics: Clarendon Press, 1991.

[38] Gelman A, Rubin DB. Inference from iterative simulation using multiple sequences. Institute of Mathematical Statistics, Statistical science, 1992, 7(4):457–472.

[39] Brooks SP, Gelman A. General methods for monitoring convergence of iterative simulations. Journal of computational and graphical statistics, 1998, 7(4):434–55. 10.1080/10618600.1998.10474787

[40] Boone EL, Merrick JR, Krachey MJ. A Hellinger distance approach to MCMC diagnostics. Journal of Statistical Computation and Simulation, 2014, 84(4):833–49. 10.1080/00949655.2012.729588

[41] Poncet P., R Core Team. statip: Statistical Functions for Probability Distributions and Regression. R package version 0.2.3., 2019.

[42] Borchers HW. Package ‘pracma’: Practical numerical math functions. R package version 2.2.9, 2019;2(1).

[43] Devore JL, Berk KN. Modern mathematical statistics with applications. Springer, 2012, 249–252.

[44] MacKay DJ, Mac Kay DJ. Information theory, inference and learning algorithms. Cambridge university press, 2003, 34–35.

[45] Gelman A, Carlin JB, Stern HS, Dunson DB, Vehtari A, Rubin DB. Bayesian data analysis. Chapman and Hall/CRC, 2013, ed. 3, 286–287.

[46] Venables WN, Ripley BD. Modern applied statistics with S-PLUS. Springer Science and Business Media, 2013, 352–355.

[47] Neal RM. MCMC using Hamiltonian dynamics. Handbook of markov chain monte carlo, 2011, 2(11):2.

[48] Kristensen K, Nielsen A, Berg CW, Skaug H, Bell B. Template model builder TMB. J. Stat. Softw, 2015, 70:1–21

