# Supplementary material for: A comparison of Monte Carlo sampling methods for metabolic network models
Source: PLoS One. 2020 Jul 1;15(7):e0235393. doi: 10.1371/journal.pone.0235393 (PMC7329079; doi:10.1371/journal.pone.0235393)
Supplement: S1 Fig — The plots are for ACHR, OPTGP and the Gibbs sampler (vertical axis) versus CHRR (horizontal axis) for six models. Sample means (v¯) (blue) and standard deviations (s) (green) are calculated according to the formulas in the manuscript. The Pearson correlation r is shown on top of each scatter plot, and the proportion of outliers removed is given in parenthesis. The sample means and standard deviations marked in red correspond to the reactions for which at least one of the two algorithms in a comparison failed the Geweke test. The identity line (pink dashed) is included to ease comparison. (PDF) [file pone.0235393.s001.pdf]

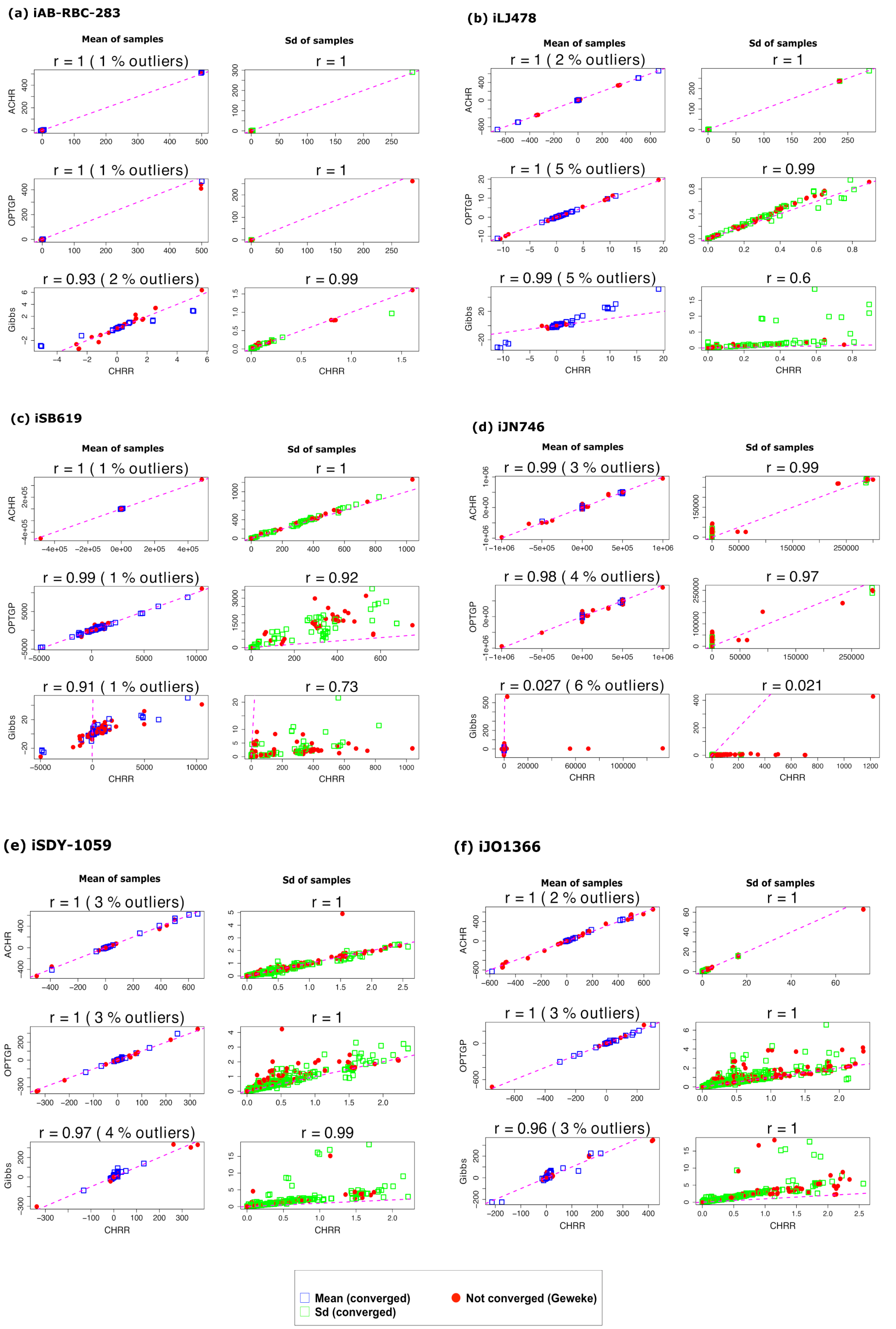

Fig S1. Scatter plot of sample means and standard deviations. The plots are for ACHR, OPTGP and the Gibbs sampler (vertical axis) versus CHRR (horizontal axis) for six models. Sample means ($\bar{v}$) (blue) and standard deviations (s) (green) are calculated according to the formulas in the manuscript. The Pearson correlation r is shown on top of each scatter plot, and the proportion of outliers removed is given in parenthesis. The sample means and standard deviations marked in red correspond to the reactions for which at least one of the two algorithms in a comparison failed the Geweke test. The identity line (pink dashed) is included to ease comparison.
